# Natural Antimicrobial Agents Utilized in Food Preservation

**DOI:** 10.3390/foods12183484

**Published:** 2023-09-19

**Authors:** Sunčica Kocić-Tanackov, Hrvoje Pavlović

**Affiliations:** 1 Faculty of Technology Novi Sad, University of Novi Sad, 21000 Novi Sad, Serbia; 2 Faculty of Food Technology Osijek, University of Osijek, 31000 Osijek, Croatia; hrvoje.pavlovic@ptfos.hr

Since the initial transformation of food surpluses, improving food quality and safety are of principal importance to human health. Due to the mass production (eggs, poultry, meat, grains, and pulses) of huge quantities of food, as well as storage and transport, food technologists are faced with challenges of contamination, undesirable microbial growth, the production of toxins, or the deterioration of food (food spoilage). Different food preserving methods (drying, smoking, etc.) have been developed throughout human history to increase the storage time of perishable raw materials or improve diets. For a long time, chemical preservatives used in the prevention of food spoilage or foodborne diseases were considered convenient for consumer protection. Effective in small concentrations and maintaining the sensory properties of food, as well as exhibiting stability at different temperatures or pH values, made their application in the prevention of food spoilage highly applicable. Indeed, they reduce food losses, increase quality, extend shelf life, and enable the development of new formulations as well as food stabilization and standardization [1]. Although consumers still demand safe, fresh-like (minimally processed), nutritionally highly valuable, high-quality foods, attitudes towards chemical conservatives have changed in contemporary nutrition. The continuous intake of chemicals during our (increasingly long-lived) lifetime and the documented adverse activity have changed consumer perceptions and attitudes towards synthetic preservatives in food technology [2]. More natural food production or the application of natural compounds in maintaining food safety have become highly desirable for many consumers in developed countries. Bio-preservatives, naturally occurring compounds from plants, animals, or microorganisms, can be successfully used in extending the shelf life of food, the inhibition/elimination of spoilage and pathogenic microorganisms, and the enhancement of food’s functionality and quality. Natural antimicrobials can be used directly in product formulation, incorporated into packaging material, or surface-coated to prevent spoilage processes or pathogen growth [3].

The most important naturally occurring used compounds in the food industry are essential oils, enzymes, and edible coatings.

Essential oils are highly volatile compounds from herbs and spices such as basil, thyme, oregano, cinnamon, clove, and rosemary, and are used to reduce spoilage microorganisms, increase overall food quality, and to inhibit food-borne pathogens such as *Salmonella*, *Listeria monocytogenes*, *Escherichia coli*, *Bacillus cereus*, and *Staphylococcus aureus* [4]. Essential oils are effective in inhibiting fungal growth as well as mycotoxin synthesis, reducing fungal damage and health risks [5,6]. Although essential oils are mainly used in the food industry as flavorings, by increasing knowledge of their modes of action and interactions with food matrix components, they can be successfully used in reducing targeted microbes. 

Enzymes from animal sources such as lyzozime, lactoferrin, and bacteriocins (natamycin, nisin, pediocin, and reuterin) from bacteria are used in small concentrations in a similar way to prolong shelf life and inhibit pathogen proliferation [7]. 

Edible coatings, thin layers of naturally occurring polymers, with or without the addition of essential oils or enzymes, used for food coating reduce moisture loss, reduce microbial contamination, and minimize the impact of packaging materials on the environment [8].

Many natural compounds are promising for replacing synthetic food additives while improving overall quality and safety. Through cooperation, food scientists and food technologists can help meet consumer needs for safe and nutritionally valuable food without the adverse effect of synthetic preservatives. 

This Special Issue aims to publish quality articles on natural antimicrobials in food preservation, their activity towards pathogens and contaminants, and novel formulations or applications in the production of safe and healthy foods. 
